# Editorial: “Thyroid Hormone in Brain and Brain Cells”

**DOI:** 10.3389/fendo.2015.00099

**Published:** 2015-06-22

**Authors:** Frédéric Flamant, Noriyuki Koibuchi, Juan Bernal

**Affiliations:** ^1^CNRS, INRA, Université de Lyon, Université Lyon 1, Lyon, France; ^2^CNRS, Institut de Génomique Fonctionnelle de Lyon, Université de Lyon, Université Lyon 1, Lyon, France; ^3^Department of Integrative Physiology, Gunma University Graduate School of Medicine, Maebashi, Gunma, Japan; ^4^Consejo Superior de Investigaciones Científicas, Center for Biomedical Research on Rare Diseases (CIBERER), Instituto de Investigaciones Biomédicas “Alberto Sols”, Universidad Autónoma de Madrid, Madrid, Spain

**Keywords:** thyroid hormones, thyroid hormone signaling, gene transcription, editorial, neurodevelopmental function of TH

Thyroid hormones (TH) function in brain has been known for a very long time. In 1813, Jean-François Coindet, a Swiss physician, made the discovery that iodine was efficient at treating goiter and cretinism, a disease associated to mental retardation, which was endemic in his country. This started a persisting tradition of research, which first identified TH [first thyroxine (T4) and then 3,5,3′-tri-iodo-l-thyronine) (T3)] as the active iodinated compounds, which early deficiency explained cretinism. It also revealed a number of other functions for TH, not only during development but also in adult brain. It is now well established that most brain cell types need TH for a proper and timely differentiation. What makes the situation in brain different than in other organs is that the consequences of TH deficiency become quickly irreversible. After the elucidation of the corresponding signaling pathway, and the identification of the two genes, now called THRA and THRB, which encode the nuclear receptors of T3 (TRs, including TRα1, TRβ1, and TRβ2), one would expect that this research field would eventually run out of unsolved mysteries. This is far from being the case and it seems that new questions keep arising all the time. This special issue is a snapshot on some of current hot topics, which bring a stimulating overview of the current situation.

One key aspect of the neurodevelopmental function of TH, bringing major complications, is that TH does not freely circulate in all brain areas and cell types, as originally postulated. TH signaling seems therefore to be heterogeneous and dynamic in brain. First of all, local metabolism by deiodinases can modulate the availability of TH. This has been exemplified in anterior cortex (1), and studied in details in inner ear (2). Second, specific transporters play a major role for the distribution of TH. Therefore, although most brain cells possess at least one of the TR, the levels of TH in serum provide little indication for the TH-signaling level in different brain areas. The physiopathological relevance of the question of TH transport in brain is best illustrated by the Allan–Herndon–Dudley syndrome, a devastating genetic disease because of a genetic mutation in MCT8, a gene encoding one of the TH transporters (3). Schroeder and Privalsky provide a clear introduction to this difficult question, which involves local metabolism of TH by deiodinases, and specific transporters required for TH to cross the blood–brain barrier or to reach the cell nucleus (4). Anticipating recent reports for the importance of non-genomic pathways for TH signaling in brain (5) they raise the hypothesis that T4 itself may be more than a prohormone, having a function different from T3 in some situations. They also explain that differential expression of coregulators may modulate TH signaling during development, a possibility that has not yet been extensively explored (6, 7). Muller and Heuer (8) provide a novel and extensive description of the main TH transporters expression patterns in mice. These new data confirm that the transporter can potentially generate a very heterogeneous distribution of TH in brain. Wirth et al. (9) provide a comprehensive overview of the growing knowledge on TH transporters in brain, in various vertebrate models. They also discuss the possibility that other iodinated compounds, which are also transported in brain, may have a neglected function, independent of the classical TR pathway. Although neurons are the primary target of T3 actions, most of the T3 present in brain is made by T4 deiodination, which takes place predominantly, if not exclusively, in glial cells: the tanycytes lining part of the third ventricle surface and in the astrocytes throughout the brain. Morte and Bernal (10) show that how the combination of primary cell cultures, genome-wide expression analysis, and mouse genetics recently revealed a dynamic evolution of this astrocytes–neurons dialog during neurodevelopment. This also led to the puzzling conclusion that the source of T3 matters: some genes, which expression is down-regulated by T3, would respond differently, depending on whether T3 crossed the brain–blood barrier, or was produced by local deiodination of T4. A general picture emerges, where TH become much more than a trophic factor, their tightly regulated distribution providing positional information to the developing neurons.

The neurodevelopmental consequences of altered TH signaling have been studied in great details over the years. Cerebellum proved to be a brain area suitable for in-depth investigation in rodent models. The first advantage, compared to other brain areas, is its relatively simple neuroanatomy, which few main cell types. The other is that its maturation takes place at a late stage of brain development, within the first post-natal weeks in rodent, when the circulating level of TH is normally high. This probably explains why the histological consequences of early TH deficiency are particularly dramatic in this brain area. Faustino and Ortiga-Carvalho review the recent progresses in our understanding in the way TH coordinate cellular interactions during this process, and the limited knowledge that we have on TRα1 and TRβ1 target genes in this promising model (11). In an ambitious reflection, Berbel et al. (12) generalize these concepts to cortex development, carefully discussing the relevance of rodent models to human pathology, and placing animal studies in an evolutionary perspective. In-depth examination of T3 regulated genes reveals hidden connection between TH deficiency and major neurodevelopmental diseases: epilepsy, autism, attention deficit hyperactive disorder, and schizophrenia. This landmark review should have far reaching consequences for later investigations, as it outlines that T3 is an essential timer of brain development, and that any alteration in T3 signaling has long-term consequences on neurological and cognitive functions. Remaud et al. (13) show that TH neurodevelopmental functions in brain do not stop after maturation, but persist throughout life, as the differentiation of adult neural stem cells, present in the hippocampus and the subventricular zone, also depends on TH. This leads to the proposal that the known decline of TH levels upon aging, may partially explain several adverse effects on cognitive functions.

One site in the adult brain where TH has been proved to exert a number of important functions is the hypothalamus. This is especially important because hypothalamus is a brain area, which communicates with peripheral organs and a place where many physiological processes can cross-talk. Some TH functions, which are thought to involve peripheral organs, may actually stem in the hypothalamus. One important example is that the control of energy homeostasis, originally believed to result from direct stimulation of liver, muscles, white, and brown adipose tissue. It is now demonstrated that this important function of TH also involves the hypothalamus, which secretes a number of signaling peptides and set the sympathetic tone (14). Three reviews focus on one hypothalamic function of TH, which is an area of intense investigations: the involvement of TH in the so-called seasonal clock, which allow many animal species to reproduce at a specific season. Using a fish model, Ogawa et al. present new data showing that TH can activate, directly or indirectly, the expression of *Kiss2* and *Gnrh* genes in hypothalamus, which are important upstream effectors of the gonadotrophic axis (15). Shinomiya et al. explain how initial studies of the photoperiodic change in gene expression in hypothalamus, performed in quail by the Nagoya group, led to the discovery of a general mechanism, common to vertebrate, which allow to couple the seasonal change in day length and reproduction (16). Dardente et al. highlight several missing links in this general model, which suggest that important contributions are still ahead (17).

All these contributions provide a timely update of an abundant literature, and suggest exciting avenues for new investigations. These will also be stimulated by new questions raised by the discovery of new genetic diseases altering TH signaling in brain (18) and by the concern that some environmental contaminants acting as TH disruptors might compromise normal brain development (19). Most of all, as TH act primarily on gene expression, studies of TH function in brain will continue to provide an outstanding opportunity to explore the basic genetic mechanisms, which govern neurodevelopment and adult brain functions.

## Conflict of Interest Statement

The authors declare that the research was conducted in the absence of any commercial or financial relationships that could be construed as a potential conflict of interest.

## References

[1] Hernandez A Quignodon L Martinez ME Flamant F St Germain DL. Type 3 deiodinase deficiency causes spatial and temporal alterations in brain T3 signaling that are dissociated from serum thyroid hormone levels. Endocrinology (2010) 151:5550–8. 10.1210/en.2010-0450 20719855 PMC2954712

[2] Ng L Hernandez A He W Ren T Srinivas M Ma M A protective role for type 3 deiodinase, a thyroid hormone-inactivating enzyme, in cochlear development and auditory function. Endocrinology (2009) 150:1952–60. 10.1210/en.2008-1419 19095741 PMC2659284

[3] Friesema EC Ganguly S Abdalla A Manning Fox JE Halestrap AP Visser TJ. Identification of monocarboxylate transporter 8 as a specific thyroid hormone transporter. J Biol Chem (2003) 278:40128–35. 10.1074/jbc.M300909200 12871948

[4] Schroeder AC Privalsky ML Thyroid hormones, t3 and t4, in the brain. Front Endocrinol (2014) 5:40 10.3389/fendo.2014.00040 PMC3978256 24744751

[5] Martin NP Fernandez de Velasco EM Mizuno F Scappini EL Gloss B Erxleben C A rapid cytoplasmic mechanism for pi3 kinase regulation by the nuclear thyroid hormone receptor, TRbeta, and genetic evidence for its role in the maturation of mouse hippocampal synapses in vivo. Endocrinology (2014) 155:3713–24. 10.1210/en.2013-2058 24932806 PMC4138568

[6] Yousefi B Jingu H Ohta M Umezu M Koibuchi N. Postnatal changes of steroid receptor coactivator-1 immunoreactivity in rat cerebellar cortex. Thyroid (2005) 15:314–9. 10.1089/thy.2005.15.314 15876152

[7] Ramos HE Weiss RE. Regulation of nuclear coactivator and corepressor expression in mouse cerebellum by thyroid hormone. Thyroid (2006) 16:211–6. 10.1089/thy.2006.16.211 16571082

[8] Muller J Heuer H. Expression pattern of thyroid hormone transporters in the postnatal mouse brain. Front Endocrinol (2014) 5:92. 10.3389/fendo.2014.00092 24994998 PMC4061481

[9] Wirth EK Schweizer U Kohrle J. Transport of thyroid hormone in brain. Front Endocrinol (2014) 5:98. 10.3389/fendo.2014.00098 25009532 PMC4067591

[10] Morte B Bernal J. Thyroid hormone action: astrocyte-neuron communication. Front Endocrinol (2014) 5:82. 10.3389/fendo.2014.00082 24910631 PMC4038973

[11] Faustino LC Ortiga-Carvalho TM. Thyroid hormone role on cerebellar development and maintenance: a perspective based on transgenic mouse models. Front Endocrinol (2014) 5:75. 10.3389/fendo.2014.00075 24904526 PMC4033007

[12] Berbel P Navarro D Roman GC. An evo-devo approach to thyroid hormones in cerebral and cerebellar cortical development: etiological implications for autism. Front Endocrinol (2014) 5:146. 10.3389/fendo.2014.00146 25250016 PMC4158880

[13] Remaud S Gothie JD Morvan-Dubois G Demeneix BA. Thyroid hormone signaling and adult neurogenesis in mammals. Front Endocrinol (2014) 5:62. 10.3389/fendo.2014.00062 24808891 PMC4009442

[14] Lopez M Alvarez CV Nogueiras R Dieguez C. Energy balance regulation by thyroid hormones at central level. Trends Mol Med (2013) 19(7):418–27. 10.1016/j.molmed.2013.04.004 23707189

[15] Ogawa S Ng KW Xue X Ramadasan PN Sivalingam M Li S Thyroid hormone upregulates hypothalamic kiss2 gene in the male Nile tilapia, *Oreochromis niloticus*. Front Endocrinol (2013) 4:184. 10.3389/fendo.2013.00184 24324459 PMC3839095

[16] Shinomiya A Shimmura T Nishiwaki-Ohkawa T Yoshimura T. Regulation of seasonal reproduction by hypothalamic activation of thyroid hormone. Front Endocrinol (2014) 5:12. 10.3389/fendo.2014.00012 24600435 PMC3930870

[17] Dardente H Hazlerigg DG Ebling FJ Thyroid hormone and seasonal rhythmicity. Front Endocrinol (2014) 5:19 10.3389/fendo.2014.00019 PMC3935485 24616714

[18] Bochukova E Schoenmakers N Agostini M Schoenmakers E Rajanayagam O Keogh JM A mutation in the thyroid hormone receptor alpha gene. N Engl J Med (2012) 366:243–9. 10.1056/NEJMoa1110296 22168587

[19] Miller MD Crofton KM Rice DC Zoeller RT. Thyroid-disrupting chemicals: interpreting upstream biomarkers of adverse outcomes. Environ Health Perspect (2009) 117:1033–41. 10.1289/ehp.0800247 19654909 PMC2717126

